# Supplementary material for: Splice-Junction-Based Mapping of Alternative Isoforms in the Human Proteome
Source: Cell Rep. Author manuscript; Available in PMC 2020 Jan 15. (PMC6961840; doi:10.1016/j.celrep.2019.11.026)
Supplement: 2 [file NIHMS1546469-supplement-2.zip › DF1/PXD010154/Liver/Liver_57_ELOVL1_GFMIVYNFSLVALSLYIVYEIPGSR.pdf]

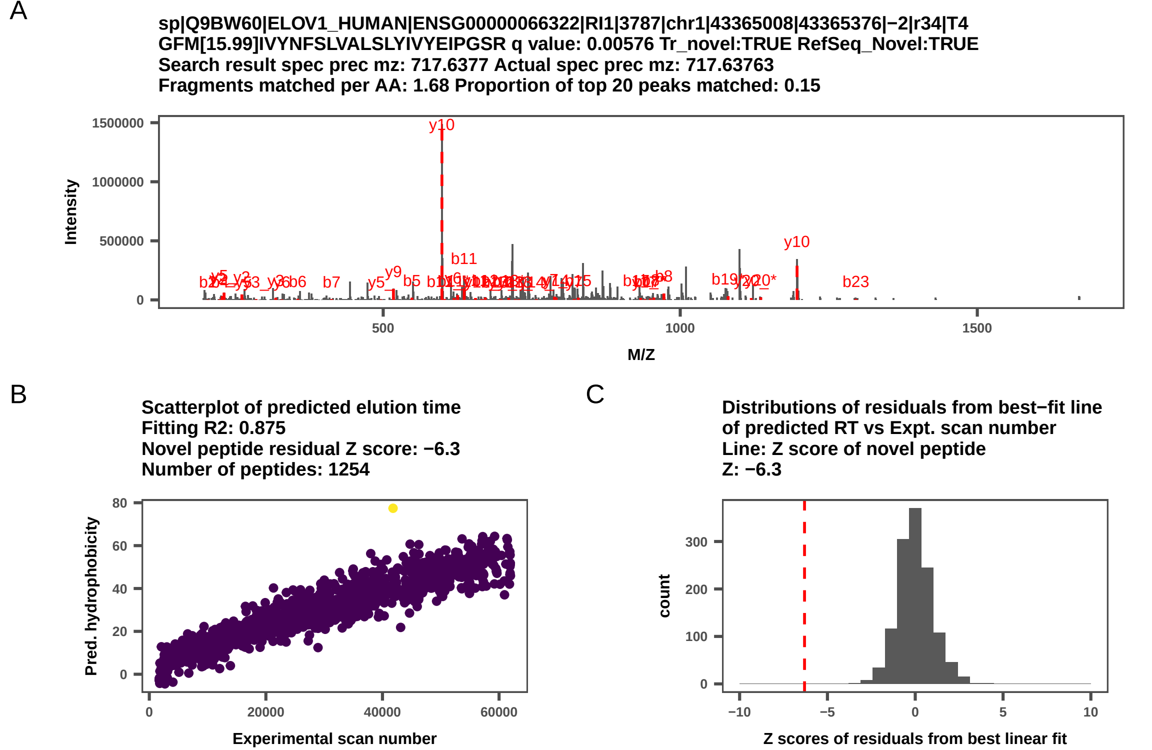
A
sp|Q9BW60|ELOV1_HUMAN|ENSG00000066322|RI1|3787|chr1|43365008|43365376|−2|r34|T4
GFM[15.99]IVYNFSLVALSLYIVYEIPGSR q value: 0.00576 Tr_novel:TRUE RefSeq_Novel:TRUE
Search result spec prec mz: 717.6377 Actual spec prec mz: 717.63763
Fragments matched per AA: 1.68 Proportion of top 20 peaks matched: 0.15
Intensity
1500000
1000000
500000
0
y10
b11
y10
b7
b5
y9
b23
500
1000
1500
M/Z
B
Scatterplot of predicted elution time
Fitting R2: 0.875
Novel peptide residual Z score: −6.3
Number of peptides: 1254
Pred. hydrophobicity
80
60
40
20
0
0
20000
40000
60000
Experimental scan number
C
Distributions of residuals from best−fit line
of predicted RT vs Expt. scan number
Line: Z score of novel peptide
Z: −6.3
count
300
200
100
0
−10
−5
0
5
10
Z scores of residuals from best linear fit
